# The value of EEG attenuation in the prediction of outcome in COVID-19 patients

**DOI:** 10.1007/s10072-022-06354-8

**Published:** 2022-08-27

**Authors:** Lucio Marinelli, Irene Pappalardo, Chiara Robba, Laura Saitta, Corrado Cabona, Denise Battaglini, Monia Casaleggio, Anna Bellini, Alessandra Ferrari, Iole Brunetti, Flavio Villani

**Affiliations:** 1 Department of Neuroscience Rehabilitation Ophthalmology, Genetics, Maternal and Child Health, University of Genova, Largo Daneo 3, 16132 Genova, Italy; 2 IRCCS Ospedale Policlinico San Martino, Division of Clinical Neurophysiology, Genova, Italy; 3 IRCCS Ospedale Policlinico San Martino, Anesthesia and Intensive Care Unit, Genova, Italy; 4 Department of Surgical Sciences and Integrated Diagnostics, University of Genova, Genova, Italy; 5 IRCCS Ospedale Policlinico San Martino, Department of Neuroradiology, Genova, Italy; 6 Department of Medicine, University of Barcelona, Barcelona, Spain

**Keywords:** SARS-CoV-2, EEG background, EEG attenuation, EEG suppression, Outcome, Encephalopathy, Hypoxemia

## Abstract

**Introduction:**

During the COVID-19 pandemic, electroencephalography (EEG) proved to be a useful tool to demonstrate brain involvement. Many studies reported non-reactive generalized slowing as the most frequent pattern and epileptiform activity in a minority of patients.

**Objective:**

To investigate the prevalence of diffuse unreactive background attenuation or suppression and its correlation with outcome in a cohort of COVID-19 patients.

**Methods:**

The EEGs recorded during the first year of the COVID-19 pandemic were retrospectively evaluated to identify the main pattern and focus on the occurrence of a low-voltage background, either attenuated (10–20 μV) or suppressed (< 10 μV). We sought a correlation between in-hospital mortality and low-voltage EEG. In a subsample of patients, biomarkers of inflammation, hypoxemia and organ failure were collected. Brain imaging was also evaluated.

**Results:**

Among 98 EEG performed in 50 consecutive patients, diffuse unreactive slowing was the most prevalent pattern (54%), followed by unreactive attenuation or suppression pattern (26%), being the latter significantly correlated with an unfavourable outcome (*p* = 0.0004). Survivors showed significantly lower interleukine-6 values compared to non-survivors. Patients with attenuated EEG and non-survivors also showed lower PaO_2_/FiO_2_ values. Neuroradiological findings were very heterogeneous with a prevalence of lesions suggestive of a microangiopathic substrate.

**Conclusions:**

EEG attenuation or suppression may be more frequent than previously reported and significantly associated with a poor outcome. SARS-CoV-2 infection may result in encephalopathy and reduced EEG voltage through mechanisms that are still unknown but deserve attention given its negative impact on prognosis.

## Introduction

Severe acute respiratory syndrome coronavirus disease (SARS-CoV-2) outbreak exerted important pressure on National Health Systems of many countries, with consequent need of increasing the number of beds for COVID-19 patients and limiting diagnostic procedures to those providing the highest benefit/risk ratio for patients and health care professionals. In this context, bedside electroencephalography (EEG) has been essential to confirm brain involvement during COVID-19 infection, helping to diagnose epileptiform activity or encephalopathic patterns in patients with impaired consciousness and, in those admitted to intensive care unit, delay in regaining it after sedation was stopped. Since the first half of 2020, many studies investigated which EEG patterns were most frequently associated with COVID-19 infection, in order to define how SARS-CoV-2 affects the brain. It has been questioned whether the virus passes the blood–brain barrier or acts indirectly through the activation of a systemic inflammatory response [1]. Other questions were related to the features of central nervous system involvement (focal or diffuse) and on how frequently epileptiform activity appears [2–6]. Many studies agree that epileptic activity may be present in about 25% of cases, while the most frequent pattern consists in non-reactive generalized slowing, occurring in up to 84% of cases [2, 4, 5, 7–15]. All case series report low percentages of patients with asymmetrical EEG patterns, focal slowing, or lateralized epileptiform activity. Diffuse voltage attenuation has been considered an infrequent pattern, with only one recent study emphasizing this condition [16]. We hypothesize that in COVID-19 patients a stereotyped EEG diffuse attenuated unreactive pattern is more frequent than previously considered. It can be related to a bad outcome, prompting the need for retrospective analysis to clarify the prognostic meaning of EEG attenuation in COVID-19 patients.

## Materials and methods

### Patient selection

This retrospective study considered consecutive electroencephalographic recordings performed between March 12, 2020, and March 16, 2021, in patients admitted to the “IRCCS Ospedale Policlinico San Martino” (Genova, Italy). EEG was performed in patients with positive molecular SARS-CoV-2 swab according to local clinical practice and experiencing different degrees of pulmonary involvement, as well as altered consciousness status, coma after the suspension of sedation or suspected status epilepticus.

### EEG recording and evaluation

All recordings were performed at patients’ bedside using subdermal needle electrodes in a reduced 8-electrodes 10–20 montage. Duration was kept at a minimum (between 10 and 20 min) in order to reduce personnel exposition to a SARS-CoV-2 contaminated environment. During the recordings, auditory (calling of patient’s name) and somatosensory/painful stimuli were applied with a minimum of 10-s intervals. EEG channels were filtered with a 1.6–70 Hz band-pass plus a 50-Hz notch filter. A single-electrocardiographic channel was simultaneously recorded. Continuous EEG monitoring was not performed in any patient for practical reasons related to the isolated environment.

The EEG tracings have been visually inspected in order to identify the main EEG pattern and evaluate the prognostic value of such a pattern. Three neurologists with expertise in EEG interpretation (LM, IP, FV) independently reviewed and classified EEG recordings according to the following patterns: (1) generalized/focal slowing, (2) rhythmic delta activity, (3) periodic patterns, (4) epileptiform discharges, (5) background voltage attenuation/suppression, (6) no abnormalities. Symmetry of EEG activity was also assessed either in terms of background frequency/amplitude or occurrence of lateralized slowing. Particular attention was devoted to background amplitude attenuation, considered as “most or all activity < 20 μV in longitudinal bipolar with standard 10–20 electrodes, measured from peak to trough” [17]. In case of disagreement among the 3 evaluators, a consensus was reached upon collegial discussion. The evaluators were blinded with respect to the patients’ outcome.

We considered attenuated EEG both recordings with background attenuation (background activity between 10 and 20 μV) and those with background suppression (activity < 10 μV). For those patients recorded more than once, only the first and the last EEG were classified and included in the analysis. In patients who performed only one EEG, the first and last EEG corresponded to the same recording.

### Outcome measures

The main outcome measures were (1) identification of EEG patterns and (2) association between attenuation or suppression pattern and in-hospital mortality.

### Blood biomarkers, clinical findings and neuroimaging data

When available, blood laboratory test including C-reactive protein, D-dimer, ferritin and interleukine-6 (IL-6) were collected in order to assess systemic inflammatory response.

The amount and duration of hypoxemia in a subgroup of patients admitted to the intensive care unit were estimated by recording the number of days with arterial partial pressure of oxygen (PaO_2_)/fraction of inspired oxygen (FiO_2_) < 300 and the lowest PaO_2_/FiO_2_ values. Similarly, organ failure assessment was assessed by the Sequential Organ Failure Assessment (SOFA) score at intensive care unit admission.

Among patients who performed brain neuroimaging, computed tomography (CT) and/or magnetic resonance imaging (MRI) were retrospectively evaluated by and expert neuroradiologist (LS).

### Statistical analysis

No sample size calculation was necessary due to the retrospective and explorative nature of our data. To recognize whether EEG attenuation could predict an unfavourable outcome in COVID-19 patients, a 2 × 2 chi-square test was performed to compare the presence of attenuation in the last EEG versus all other patterns in patients who died versus those who survived.

Blood laboratory tests and clinical finding values were compared between patients with and without EEG attenuation in the last EEG and between survivors and non-survivors using the Mann–Whitney *U* test.

All data are reported as median and first-third interquartile range. The significance level was set at *p* < 0.05. All study procedures were performed according to the Declaration of Helsinki.

## Results

In the considered 12-month time window during COVID-19 first-wave pandemic, 98 electroencephalographic recordings were performed in 50 consecutive all-Caucasic patients (Table 1). The main reasons for EEG request were an unexplained impairment of consciousness with or without history of epileptic seizures or difficult awakening/altered consciousness after the sedation was stopped, in the absence of major abnormalities on blood tests and/or neuroimaging that could explain the patient’s status. Most patients were directly admitted at the Emergency Department of the San Martino hospital (48, 96%), whereas 2 (4%) were centralized from other hospitals of Liguria region. When the first EEG was recorded, 31 (62%) patients were admitted to an intensive care unit.

**Table 1:** Patient demographic data

	Number	Gender (M/F)	Age
Total	50	32/18	66 (59–74)
Deceased	29	20/9	68 (63–72)
Survived	21	12/9	63 (58–75)

Age is reported as median (1st–3rd quartiles)

**Fig. 1:**
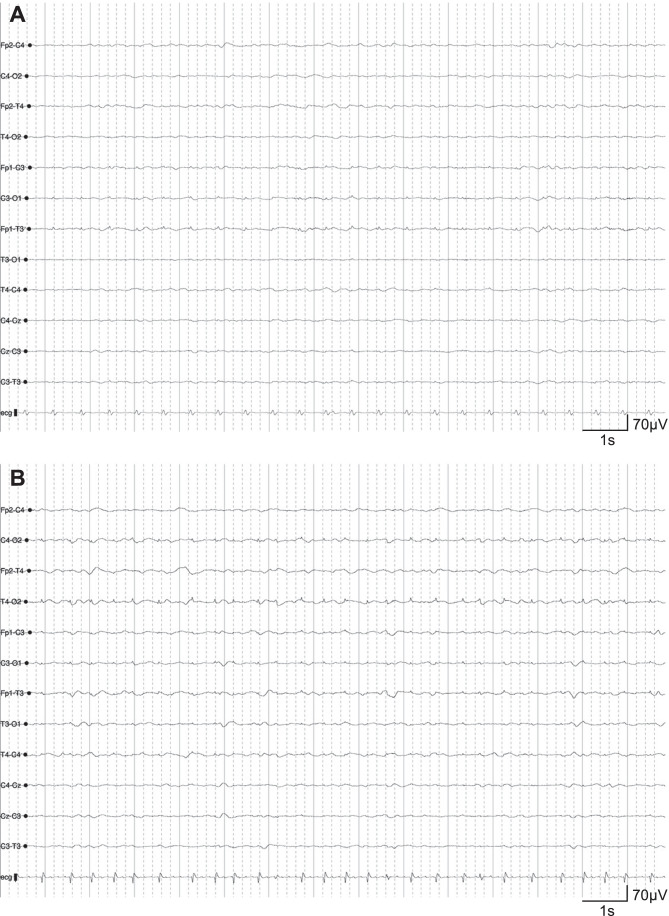
Illustrative attenuated EEG recording. **A** Attenuate EEG recording from a non-survivor 65-year-old male. **B** Attenuate EEG recording from a non-survivor 73-year-old female

The prevalent EEG pattern was diffuse slowing (*n* = 27, 54%). Epileptiform activity was detected only in 6 patients (12%), while normal findings emerged in 3 (6%). One patient presented rhythmic delta pattern (*n* = 1, 2%). Attenuation was the second most prevalent pattern with 13 cases (26%) (Fig. 1). No periodic patterns have been detected.

In 27 patients, EEG was recorded once, in 23 cases more than once. All patients had symmetrical pattern apart from 1 who had an ischaemic stroke and lateralized epileptiform discharges were recorded ipsilateral to the lesion.

**Fig. 2:**
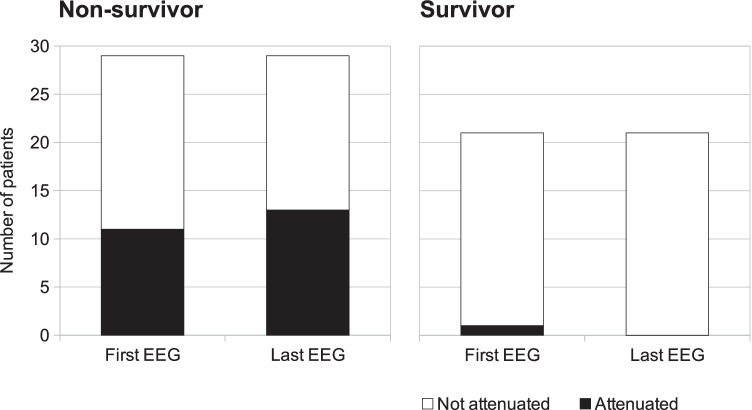
Attenuated EEG pattern is prevalent in non-survivors. Among the 29 patients who did not survive, many EEG were attenuated (first EEG: 11 last EEG: 13; left panel), while in the 21 patients who survived, only 1 EEG was initially attenuated, but eventually it no longer was, so that at the last EEG recording all survivors had a not attenuated EEG (right panel)

The first EEG was attenuated in 12 (24%) patients: 1 among those who survived and 11 among those who died. The last EEG was attenuated in 13 (26%) patients, and none survived. The patient with attenuated first EEG who survived had the last EEG no longer attenuated. Conversely, the 2 deceased patients who had a not-attenuated first EEG developed the attenuated pattern at the last recording (Fig. 2). Patients with unfavourable outcome had more frequently attenuated EEG (*χ*^2^(1, *N* = 50) = 12.7, *p* = 0.0004). Among the 50 patients of our series, 29 did not survive, reflecting a 58% mortality.

Laboratory tests failed to find significant difference between patients with attenuated/not-attenuated EEG (Table 2), while survivors showed significantly lower IL-6 values when compared to non-survivors (Table 3). Clinical findings related to hypoxemia and organ failure revealed that patients with attenuated EEG had lower PaO_2_/FiO_2_ values than those without an attenuated EEG, while the number of days with PaO_2_/FiO_2_ < 300 and the SOFA scores did not differ (Table 2). Similarly, non-survivors had lower PaO_2_/FiO_2_ values than non-survivors, without significant difference concerning the number of days with PaO_2_/FiO_2_ < 300 and the SOFA scores (Table 3).

**Table 2:** Laboratory/clinical findings and EEG attenuation

Laboratory test	*N*	Mean ± SD	Mann–Whitney *U*	*p*
C-reactive protein (mg/l)			19	0.2
Not attenuated	9	279 ± 84		
Attenuated	9	218 ± 145		
D-dimer (µg/l)			57	0.9
Not attenuated	13	7918 ± 9529		
Attenuated	9	16,285 ± 24,069		
Ferritin (µg/l)			50	0.7
Not attenuated	14	2454 ± 2914		
Attenuated	8	2349 ± 1663		
IL-6 (ng/l)			41	0.3
Not attenuated	14	139 ± 149		
Attenuated	8	133 ± 242		
Clinical findings	N	Mean ± SD	Mann–Whitney *U*	*p*
Days with PaO_2_/FiO_2_ < 300			59	0.4
Not attenuated	15	40.6 ± 20.4		
Attenuated	10	78.8 ± 71.8		
Lowest PaO_2_/FiO_2_ values			34	**0.02**
Not attenuated	15	93.5 ± 32.3		
Attenuated	10	64.5 ± 12.4		
SOFA score at admission			62	0.5
Not attenuated	15	4.4 ± 1.5		
Attenuated	10	4.9 ± 1.5		

Reference values: C-reactive protein 0–5 mg/l, D-Dimer 0–500 µg/l, Ferritin 30–400 µg/l, IL-6 0–3.4 ng/l

Significant *p* values are reported in bold

**Table 3:** Laboratory/clinical findings and outcome

Laboratory test	*N*	Mean ± SD	Mann–Whitney *U*	*p*
C-reactive protein (mg/l)			8	0.05
Survived	4	327 ± 60		
Deceased	12	217 ± 127		
D-dimer (µg/l)			44	0.4
Survived	8	4693 ± 3852		
Deceased	14	15,139 ± 20,451		
Ferritin (µg/l)			45	0.5
Survived	8	3247 ± 3669		
Deceased	14	1942 ± 1439		
IL-6 (ng/l)			19	**0.01**
Survived	8	65 ± 87		
Deceased	14	235 ± 204		
Clinical findings	*N*	Mean ± SD	Mann–Whitney *U*	*p*
Days with PaO_2_/FiO_2_ < 300			45	0.1
Survived	15	36.2 ± 21.8		
Deceased	10	66.9 ± 58.7		
Lowest PaO_2_/FiO_2_ values			33	**0.03**
Survived	15	100.8 ± 33.3		
Deceased	10	71.3 ± 21.9		
SOFA score at admission			65	0.7
Survived	15	4.3 ± 1.4		
Deceased	10	4.8 ± 1.5		

Reference values: C-reactive protein 0–5 mg/l, D-dimer 0–500 µg/l, Ferritin 30–400 µg/l, IL-6 0–3.4 ng/l

Significant *p* values are reported in bold

In our series of 50 patients, 16 patients were evaluated with magnetic resonance imaging (MRI) of the brain, while 25/50 underwent only a CT examination. Among the included patients, 9 of them never underwent a neuroradiological examination due to several reasons, including the potential risk of spreading the infection and the unstable clinical conditions. Five out of 13 patients with an attenuated EEG pattern underwent a MRI, whereas 37 had no available imaging or underwent only CT scans which were unremarkable. Among the 5 patients who performed a brain MRI, 4 of them presented an attenuated EEG pattern showed evidence of GRE T2*/SWI punctuate hypointensities; one of them showed a pattern of diffuse symmetric hyperintensity of the white matter on T2-weighted and fluid-attenuated inversion recovery (FLAIR) sequences, with evidence of a peripheral lining of restricted diffusion in DWI. The other MRI patient showed small areas of restricted diffusion on DWI. Three patients had a combination of these two entities (DWI and SWI lesions).

## Discussion

We report the experience of one of northern Italy’s largest hospitals, during a 1-year time span. In our series, diffuse attenuation pattern was common and significantly more frequently detected in patients with poor outcome. In comparison with other studies, we observed higher mortality (58%) and a high prevalence of attenuated EEG patterns. Among published data, the study by Pellinen et al. reported mortality reaching 44%, although this may have been underestimated since 20% of patients were still hospitalized when the data were analysed [13]. In our series, data entered the analysis only when all included patients were discharged or deceased. This could have contributed to the high mortality, along with the elevated median age (66 years). It must be underlined that Liguria is the Italian region with the highest percentage of elderly people and this could have been the main reason for such a high mortality. It must also be considered that included patients belonged to the first COVID-19 wave: many were probably very fragile, and vaccines were not yet available.

Diffuse attenuation has also been reported in other case series, but almost always in small percentages [2, 7, 18] or presumably none [13, 14]. Higher prevalence of attenuated patterns was reported in two more recent studies, reaching 21–25% of cases [16, 19]. The association between generalized attenuation and higher mortality was postulated but not confirmed [16]. In patients who performed more than one EEG, we observed that the attenuation pattern found in the latter EEG was more likely to predict unfavourable outcome. In fact, one patient who had an attenuated first EEG evolved in a non-attenuated pattern and survived. Conversely, two patients progressed to diffuse attenuation during follow-up and did not survive. These observations point out that EEG may have a role in predicting outcome in COVID-19 patients and multiple examinations could reflect disease progression.

Even if the majority of studies report diffuse slowing as the most frequent EEG pattern, this can be hardly considered specific for COVID-19 and the possible effect of sedation has been considered [2]. In a recent meta-analysis, the pooled prevalence of slow background abnormalities (theta and delta) in patients outside intensive care units (ICU) was 0.92 (95%CI 0.83–1.01, *I*^2^ = 68.81%). The pooled prevalence of abnormal background in non-ICU patients was 0.95 (95%CI 0.88–1.09, *I*^2^ = 44.98%) [20]. Diffuse slowing is the most frequent pattern also among our patients.

Despite the limited application of neuromonitoring tools outside the ICU during the COVID-19 pandemic, EEG findings revealed various abnormalities among non-critically ill patients with COVID-19 who manifested new neurological symptoms. The most common finding was the presence of abnormal background activity, followed by slow background, rhythmic and periodic discharges, and electroencephalographic seizures. This may be explained by various factors: (1) patients with COVID-19 might be at higher risk of hypoxic and metabolic changes responsible for encephalopathy; (2) after the virus enters the cells, a strong inflammatory response followed by cytokine storms may alter cerebral permeability and hemodynamic, thus favouring encephalopathy and multiple organ failure with potential for EEG alterations; (3) seizures, although a prevalence comparable to the non-COVID-19 population may be indicative of new neurological complications [20].

We did not systematically record patient ongoing therapy, but sedation (e.g., propofol and midazolam) was usually suspended several hours before performing EEG. Indeed, a marginal role of sedation is possible in determining generalized slowing; however, in our experience, sedation usually does not determine generalized attenuation. Burst-suppression patterns can be observed during initial sedation, with diffuse suppression occurring only during extreme sedative regimen that were not adopted in any of our cases.

Occurrence of seizures or epileptiform abnormalities was rather low in our series (*n* = 6, 12%), in line with other studies [2, 7, 13–15, 19]. However, it must be acknowledged that short recording duration could have underestimated the rate of seizures and epileptiform activity [21], even if this limitation is partially compensated by higher recording repetition compared to the other studies.

The only patient with asymmetric EEG had a focal lesion due to ischaemic stroke; therefore, our observations confirm that during COVID-19 EEG patterns are often symmetrical. Current interpretations indicate that diffuse alterations at the EEG are related to a diffuse cortical involvement due to a generalized inflammatory response, while direct invasion of central nervous system by SARS-CoV-2 is considered unlikely [4, 5, 12]. Our data favour this interpretation as the finding of increased proinflammatory IL-6 levels in patients with the worst outcome. It could be hypothesized that systemic inflammation could have been related to the diffuse attenuation pattern; however, to our knowledge, this association has never been demonstrated. Furthermore, it is known that attenuation/suppression patterns can be seen in many encephalopathic conditions, not necessarily inflammatory. Importantly, patients with an attenuated EEG and non-survivors had lower PaO_2_/FiO_2_ values, suggesting a more severe respiratory system involvement and hypoxemia, possibly reflecting on brain function.

Transitory generalized attenuation of EEG signal has been described in adults as “spontaneous intermittent generalized attenuations” (SIGA). They are brief in duration (1300 ms on average) and often associated with triphasic waves, being associated with encephalopathies and poor outcome [22]. Generalized unreactive attenuation may suggest cortical generalized injury or transitory dysfunction, being often associated with extensive cortical and subcortical lesions such as anoxic encephalopathy or severe head trauma. Existing literature suggests that generalized EEG attenuation can also be caused by reduced synchronicity of the cortical activity or by an increased distance between the cerebral cortex and recording electrodes such as in the case of the interposition of fluids or increased tissue thickness [23], either conditions unlikely in our patients. Technical factors must also be considered, especially to explain the extreme variability of diffuse attenuation patterns in published studies. Indeed, EEG waveform amplitude may depend on inter-electrode distance: waveform amplification occurs when comparing the voltage of more distant electrodes. Therefore, a reduced montage, like the one we have used, could have artificially increased waveform amplitude with respect to a standard 10–20 montage, used for international definition of attenuation [17]. Our interpretation of attenuation is therefore probably more conservative since waveform amplitude would have been even more reduced if recorded with a standard 10–20 montage.

The neuroradiological findings were very heterogeneous, though specific patterns, namely lesions suggestive of a microangiopathic substrate prevailed, in line with the findings described in COVID-19 patients with neurological complications [24]. GRE T2*/SWI punctate lesions, which tend to have a predilection for the corpus callosum, prevailed among the 5 patients with MRI and attenuated EEG. Such pattern, suggestive of microthrombosis, has been frequently described in COVID-19 patients on ventilator support, with high D-dimer levels and disturbance of consciousness, and was associated with a worse prognosis [25, 26]. Radiological/histopathological correlations support the occurrence of brain microvascular damage in COVID-19 patients [26]. In some of our patients, coexistence of small ischaemic lesions with SWI lesions is suggestive of a shared microangiopathic substrate [24]. At MRI, we also observed a leukoencephalopatic pattern, which has also been described as a possible sequela of hypoxemia in COVID-19 patients. The mechanism underlying the white matter damage is not clear. It is not distinctive of COVID-19, yet it has been described in other patients with prolonged and profound hypoxemia associated with SARS-CoV-2 infection. Neurotropism cannot be excluded as another possible cause of leukoencephalopathy [27].

This study suffers from some limitations. Because of the difficulties related to the first wave of the pandemic and the retrospective design of the study, patient clinical details and history were often incomplete and precise information about ongoing therapy was lacking and its influence on background activity was difficult to foresee. To avoid unnecessary personnel infection risk, EEG was probably not prescribed to patients with less severe neurological conditions, with a consequent probable selection bias favouring patients with worse neurological status. Furthermore, during the first wave of the pandemic, we noticed a significant reduction in COVID-unrelated hospital admissions and requests for neurological consultations in the emergency room. This could have caused a selection bias related to underdiagnosing other neurological conditions.

In conclusion, our study underlines that diffuse attenuation may be a frequent EEG pattern in COVID-19 patients, and we demonstrate for the first time that EEG attenuation is related to an unfavourable outcome.
